# Understanding the Thermal Degradation Mechanism of High-Temperature-Resistant Phthalonitrile Foam at Macroscopic and Molecular Levels

**DOI:** 10.3390/polym15193947

**Published:** 2023-09-29

**Authors:** Xulin Yang, Yi Li, Wenwu Lei, Zhongxiang Bai, Yingqing Zhan, Ying Li, Kui Li, Pan Wang, Wei Feng, Qi Liu

**Affiliations:** 1 School of Mechanical Engineering, Chengdu University, Chengdu 610106, Chinaliying@cdu.edu.cn (Y.L.);; 2 Chemical Synthesis and Pollution Control Key Laboratory of Sichuan Province, China West Normal University, Nanchong 637009, China; 3 Institute for Advanced Study, Chengdu University, Chengdu 610106, China; 17380140172@163.com; 4 Chengdu Hongbo Industrial Co., Ltd., Chengdu 610199, China; 5 College of Chemistry and Chemical Engineering, Southwest Petroleum University, Chengdu 610500, China

**Keywords:** phthalonitrile, foam, thermal degradation, degradation behavior, degradation mechanism

## Abstract

Polymer foam, a special form of polymer, usually demonstrates some unexpected properties that rarely prevail in the bulky polymer. Studying the thermal degradation behavior of a specific polymer foam is important for its rational design, quick identification, objective evaluation, and industrial application. The present study aimed to discover the thermal degradation mechanism of high-temperature-resistant phthalonitrile (PN) foam under an inert gas atmosphere. The macroscopic thermal decomposition of PN foam was carried out at the cost of size/weight loss, resulting in an increasing number of open cells with pyrolyzation debris. Using the TGA/DTG/FTIR/MS technique, it was found that PN foam involves a three-stage thermal degradation mechanism: (I) releasing gases such as H_2_O, CO_2_, and NH_3_ generated from azo-containing intermediate decomposition and these trapped in the closed cells during the foaming process; (II) backbone decomposition from C-N, C-O, and C-C cleavage in the PN aliphatic chain with the generation of H_2_O, CO_2_, NH_3_, CO, CH_4_, RNH_2_, HCN, and aromatic gases; and (III) carbonization into a final N-hybrid graphite. The thermal degradation of PN foam was different from that of bulky PN resin. During the entire pyrolysis of PN foam, there was a gas superposition phenomenon since the release of the decomposition volatile was retarded by the closed cells in the PN foam. This research will contribute to the general understanding of the thermal degradation behavior of PN foam at the macroscopic and molecular levels and provide a reference for the identification, determination, and design of PN material.

## 1. Introduction

When referring to high-temperature-resistant polymers, phthalonitrile (PN) can never be overemphasized. PN resin is defined as a class of compounds containing a cross-linkable phthalonitrile unit (CN-Ar-CN), which can be cured into heterocyclic phthalocyanine [1]. Due to the high aromaticity of cured products [2,3], PN-based materials show extremely superior temperature, heat, or fire-retardant properties, i.e., excellent thermal stabilities [4,5], high char residues [6,7], and high glass transition temperatures [8,9]. For example, a high *T*_5%_ close to 500 °C was reported in PN self-composite [4]. A high char residue of 70% was realized in a PN/CNT nanocomposite [6], and PN could be used as an early fire detection sensor [7]. The glass transition temperature of a PN/halloysite nanotube composite was as high as 380 °C [9]. Various PN resins with regulatable properties can be synthesized via nucleophilic substitution from diphenols and 4-nitrophthalonitrile under catalytic conditions [1]. PN resins are commonly processed into composite laminates [10,11], adhesives [12], fibers [13], and coatings [14] for applications in aerospace, automotive, military, and electrical industries [15].

The initial advantage of utilizing PN resin as a polymer matrix in high-performance composites is its superior high-temperature-resistant characteristic [16,17,18]. PN/fiber composites exhibit excellent comprehensive performances with little attenuation in mechanical properties after high-temperature aging [19,20,21,22]. It was reported that PN/basalt fiber laminate exhibited an impact strength of 108 kJ/m^2^ [19]. Retention of 90% of the flexural modulus was observed in PN/glass fiber/microsilica laminate after 400 °C/12 h aging [20]. At 500 °C, the flexural modulus of PN/carbon fiber composite was still retained at 80% in comparison with that at room temperature [21], and PN/carbon fabric composite showed outstanding mechanical properties at 400 °C [22]. PN/titania coating demonstrated a high initial decomposition temperature of up to 480 °C and excellent corrosion protective properties on a steel plate [23]. In view of the high char yield, Laskoski and Lei et al. [24,25] reported a method of direct solid-phase conversion of PN into carbon nanotubes, while porous carbons or aerogels have been prepared from PN precursors with high yields for electrochemical [26] or electromagnetic applications [27,28]. In the case of PN foam [29,30], a burning test over 400 °C showed no visible smoke or softening or droplet phenomena, and the macroscopic structure of the PN foam was well maintained. PN was also employed as a stabilizer for PAN fibers, and an obvious increase in char yield at 600 °C from 57.7% in PAN/PN fiber (10 wt% PN) to 69.0% in pristine PAN fiber was realized [31]. This research has proven the superior thermal stabilities of PN-based materials [19,20,21,22,23,24,25,26,27,28,29,30,31]; however, limited attention has been paid to the thermal degradation mechanisms of PN resins. As we know, studying the thermal degradation behavior of a specific polymer is very important for rational design, quick identification, objective evaluation, and industrial application [32,33].

On the other hand, Liang et al. [34] first reported the pyrolysis process of resorcinol-based PN resin, and it showed two consecutive and overlapping degradation stages with an activation energy of 347.4 kJ/mol and 329.8 kJ/mol, respectively. In later work, it was found that the main volatiles of resorcinol-based PN were H_2_O, NH_3_, HCN, CH_4_, CO_2_, and CO during its thermal degradation [35]. Zhou et al. [36] reported that L-tyrosine-based PN involved three pyrolysis stages: I (403–490 °C; CO_2_, CO, CH_4_, etc.), II (490–600 °C, NH_3_, and HCN) and III (600–1000 °C and H_2_). Guo et al. [37] revealed a four-stage pyrolysis behavior of adenine-based PN: I (oligomer volatilization, 400–500 °C), II and III (major degradation, 500–820 °C, CH_4_, NH_3_, H_2_O, CO, CO_2_, HCN, and aromatics) and IV (carbonization, 600–1000 °C, CH_4_ and NH_3_). From these advances [34,35,36,37], it is clear that PN with different structures displays different thermal degradation characteristics from two-stage [35], three-stage [36], and even four-stage [37], and the volatiles during PN dissociation have been well characterized. Nonetheless, previous studies on thermal degradation are limited to resorcinol [34,35], L-tyrosine [36], or adenine-based PN [37], and the pyrolysis behavior of other PN resins remains unknown. Specifically, these studies have concentrated on the pyrolysis of bulky PN polymers [34,35,36,37], leaving unknown territory for the thermal degradation behaviors of PN foams. During the aging of the phosphorous PN, Lobanova et al. [38] found that the weight loss rate in bulky PN samples was slower than that in PN powders due to a lower surface-to-mass ratio, indicating that the thermal decomposition behavior is tightly related to the sample form. Foam is an important and special form of a polymer since the gas phase may hinder heat transfer and thus retard thermal decomposition [39,40,41]. In general, the thermal degradation of a material can be viewed as the earliest solid combustion, and the resulting flammable volatiles promote the combustion [42]. In this sense, pores in PN foam may have a large effect on the pyrolysis behavior. 

As discussed above, for the first time, the current study was dedicated to discovering the thermal degradation mechanism of a benzoxazine-containing PN foam from macroscopic and microscopic perspectives. PN foam after thermal pyrolysis was carefully characterized to provide a general understanding of the thermal degradation behavior of the PN foam. Through the TGA/DTG/FTIR/MS technique, the real-time volatiles in the thermal degradation of PN foam were monitored. According to these results, the possible thermal degradation mechanism of PN foam was finally deluded at the macroscopic and molecular levels. This work will not only expand our cognition of the decomposition behavior of benzoxazine-containing PN resin but also help us understand the macroscopic and microscopic thermal degradation mechanism of PN foam. The current findings are of great significance for the rational design, rapid identification, objective evaluation, and industrial application of porous PN materials. This study also provides references for thermal degradation analyses of other high-temperature resistant polymer foams.

## 2. Experimental

### 2.1. Materials

**Figure 1:**
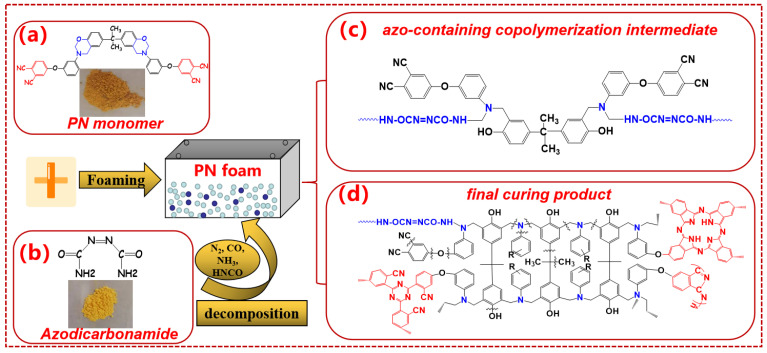
Chemical structure of (**a**) PN monomer, (**b**) azodicarbonamide, (**c**) azo-containing copolymerization intermediate [29], and (**d**) final curing product [4].

Gold benzoxazine-containing PN powder (Figure 1a) was provided by Shunde Great New Materials Co., Ltd. (Foshan, China). Yellow azodicarbonamide powder (Figure 1b) was obtained from Chengdu Kelong Chemical Co., Ltd. (Chengdu, China). 

### 2.2. Preparation

PN foam was prepared according to previous reports with minor modifications [29,30]. In this study, deionized water (disperse medium) and Tween-80 (foam stabilizer) were avoided to prevent small molecules from interfering with the pyrolysis analysis. Herein, the PN foam in this study was obtained by a direct melting–mixing process, as shown in Figure 1. After the melting of PN resin at 180 °C, azodicarbonamide (foam agent) was added and mixed for 15 min. It was then delivered to a steel mold and foamed in an oven at 180 °C. At this time, bubble growth and bubble stabilization happened simultaneously [29]. Bubble growth was caused by the decomposition of azodicarbonamide, as shown in Figure 1b. The curing of bi-functional PN resin stabilized the resulting bubbles. The curing mechanism of benzoxazine-containing PN has been well-reported by other studies [4,5]. The final curing product of the oxazine ring was mainly a tertiary amine (NR_3_) compound, while that of phthalonitrile was N-containing aromatic heterocycles, including phthalocyanine, triazine, and isoindole rings [4,5], as shown in Figure 1d. After post-curing at 200 °C for 2 h, PN foam was finally obtained and labeled as F200. To study the thermal degradation of the PN foam at the macro level, F200 was further heated to 400 °C, 600 °C, 800 °C, and 1000 °C at 2 °C/min and kept for 1 h under ab N_2_ atmosphere. For simplicity, after thermal pyrolysis, the PN foams were labeled F400, F600, F800, and F1000, correspondingly.

### 2.3. Characterization

Digital photos of the PN foams were taken by an IMX 600 camera (F1.8) on a HUAWEI P20 Pro cellphone sourced from Chengdu Huawei Technology Co., Ltd. (Chengdu, China). X-ray diffraction (XRD) of the original PN foam was performed on a Bruker D8 by CuKα radiation device. Raman spectra (RS) of various foams were conducted on a Thermo Fisher DXR2xi (Thermo Fisher Scientific Inc., Waltham, MA, USA) at 532 nm with 1 cm^−1^ resolution. The linear or volume shrinkage of the PN foam was calculated by the average ratio of difference divided by the initial value of length or volume of 3 samples before/after thermal degradation. The densities of the PN foams were investigated according to ISO 845:2006 [43]. Scanning electron microscopy (SEM) of the PN foam was conducted on a JSM-5900LV at 20 kV after gold sputtering. Thermogravimetric analysis (TGA) of the original PN foam was carried out by a TG-50 TA instrument at 20 °C/min in N_2_. TGA–FTIR of the PN foam was conducted on a TGA system (STA 449F3, NETZSCH company, Germany) accompanied by a Bruker Tensor 27 FTIR system (Bruker Optics, Inc., Billerica, MA) in argon. TGA–MS of the PN foam was tested on a mass spectrometer (Rigaku, Thermo plus EV2, Japan) from Japanese Neo Confucianism in argon.

## 3. Results and Discussions

### 3.1. Macroscopic Thermal Degradation Behavior of PN Foam

**Figure 2:**
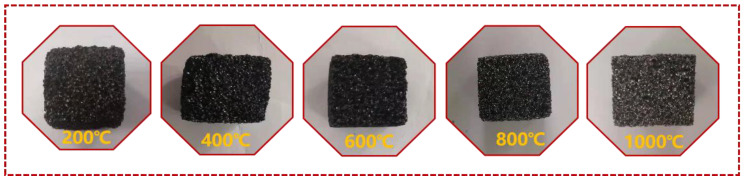
Digital photos of PN foam after thermal degradation.

**Figure 3:**
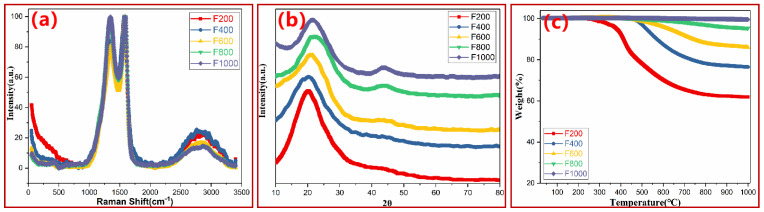
(**a**) Raman spectra, (**b**) XRD pattern, and (**c**) TGA curve of PN foam after thermal degradation.

To observe the thermal decomposition behavior of PN foam at the macro level, the original PN foam (F200) was pyrolyzed at 400 °C, 600 °C, 800 °C, and 1000 °C for 1 h. The digital photos of the PN foams after thermal degradation are displayed in Figure 2. F200 exhibited a black porous feature. As the pyrolysis temperature increased, the porous structure was well maintained, indicating the high-temperature-resistant characteristics of PN foams. Moreover, PN foam gradually showed a metallic luster like graphite [28], suggesting a process of continuing pyrolysis to further graphitization with increasing temperature, as further confirmed by Figure 3a–c. 

As shown in Figure 3a, the D band around 1350 cm^−1^ is attributed to the sp^3^ configuration, which is deemed as defects in carbon, while the G band at 1590 cm^−1^ is a reflection of the sp^2^ carbon, which implies the ordered structure of graphite [26]. Generally, graphitization extent is evaluated by the I_D_/I_G_ ratio [44,45]. In the case of the PN foam, the overall trend of the I_D_/I_G_ ratio increased with the pyrolysis temperature (Table S1), indicating a higher graphitization extent at higher temperatures, as further reflected in the XRD pattern in Figure 3b. The diffraction peak around 21° corresponds to d002 of amorphous carbon in the PN foam [26,27,28]. As the pyrolysis temperature increased to 600 °C, this peak gradually shifted to higher values, and meanwhile, a new peak at 44° corresponding to d101 of hexagonal graphite became obvious (JCPDS card no. 75-1621) [44]. A similar phenomenon has also been reported in previous references [44,45,46] and was explained by the increasing graphitization degree at higher temperatures. The full width at half maximum (FWHM) is also provided in Table S2. It was found that the FWHM of the PN foam gradually increased with the increase in temperature. Generally, for carbon materials, a smaller FWHM value in XRD means a larger grain size and fewer defects in the carbon [44,45]. Combined with the above results, it can be concluded that in PN foam, as the heat treatment increases, the graphitization degree increases, but the defects increase and the size of crystallite carbon decrease. As shown in the TGA curves shown in Figure 3c, with increasing temperature, continuous weight loss happened in F200, F400, and F600, but a high char residue over 95.4% indicated a high graphitization extent in F800 and F1000. 

**Figure 4:**
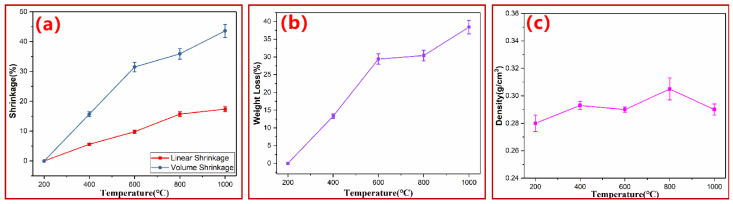
(**a**) Shrinkage, (**b**) weight loss, and (**c**) density of PN foam after thermal degradation.

At the macro level, the pyrolysis of PN foam into hybrid graphite came at the cost of size and weight loss. As shown in Figure 4a,b, both volume shrinkage and weight loss of the PN foam increased with increasing degradation temperature, suggesting that the macroscopic decomposition of the PN foam was carried out from the surface to the inside. As a result, all PN foams showed a similar density of about 0.29 g/cm^3^ (Figure 4c). 

**Figure 5:**
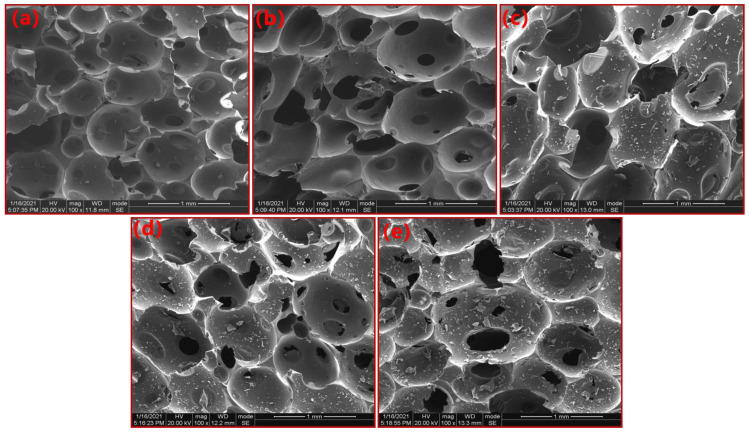
SEM images of PN foams after thermal degradation: (**a**) F200, (**b**) F400, (**c**) F600, (**d**) F800, and (**e**) F1000.

The SEM images of the PN foams after thermal degradation are provided in Figure 5. As shown in Figure 5a, the pores in F200 were spherical, including big bubbles surrounded by small cells. On the surface of some cells, circular holes could be observed. Some holes were open, while most of the holes were enclosed by a lid-like thin resin layer. By careful distinguishing, the bubbles in the PN foam can be classified into four types: fully open cell, open cell with lid, closed cell with lid, and fully closed cell (as illustrated in Figure S1). Overall, the bubbles in F200 were mainly the closed cells. As shown in Figure 5b, the four types of cells above could be also observed in F400, but more obvious were the open cells, suggesting that some decomposition gases had rushed out from the cells. As can be observed in Figure 5c–e, the contour of the bubble was maintained in F600, F800, and F1000. However, bubbles were covered with some flour-like debris, which resulted from the deposition of decomposition products. As the degradation temperature increased, there was more and more debris in the PN foams due to the severe decomposition at higher temperatures. In addition, deep holes (considered defects) could be observed, which are attributed to the diffusion and penetration of the pyrolysis volatile in the foam architecture. Nevertheless, the closed bubbles could be still observed in F600, F800, and F1000, suggesting that some decomposition volatiles were blocked inside the cell. 

To conclude, the macroscopic thermal degradation behavior of PN foam is a continual graphitization process at the cost of size/weight loss due to the release of volatiles, resulting in an increasing number of open cells with decomposition debris. Specifically, the existence of closed bubbles had a large effect on the pyrolysis behavior of the PN foam due to its barrier effect on the release of decomposition products. These results provide a general understanding of the thermal degradation behavior of PN foam at the macro level; nevertheless, its thermal degradation mechanism needs to be further clarified at the molecular level. 

### 3.2. General Thermal Degradation Behavior of PN Foam by TGA–DTG

**Figure 6:**
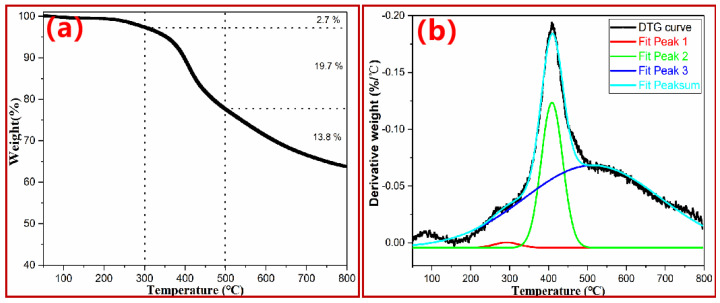
(**a**) TGA and (**b**) DTG curve of original PN foam (F200).

TGA/DTG is an effective way to characterize the real-time relationship between weight and temperature and thus provide the general thermal degradation information of the PN molecular structure. As shown in Figure 6a, the weight loss in the TGA curve can be roughly divided into three stages: the first slight dissociation happened before 300 °C, with a weight loss of 2.7%; the second was apparently the major pyrolysis of the PN foam, resulting in a 19.7% weight loss; and the third, which occurred over 650 °C, showed a weight loss of 13.8%. The PN foam finally exhibited a high char yield of 63.8% at 800 °C, which is close to the data of its bulky form [5,8].

In the DTG curves in Figure 6b, three obvious peaks confirm that PN foam has three-stage thermal degradation characteristics. However, only two pyrolysis peaks were reported in the bulky PN resin [5,8]. The maximum decomposition temperatures (*T_dm_*s) of the latter two peaks (410 °C and 519 °C) in the PN foam were very close to the *T_dm_*s of the bulky PN resin (415 °C and 520 °C) [5,8]. Herein, the latter two peaks can be attributed to the pyrolysis of the polymer backbone and further carbonization, respectively [5,8]. In this sense, the extra peak at 294 °C is worth careful consideration. The primary suspect is the residue of the blowing agent (azodicarbonamide) in the PN foam. However, the present dosage of azodicarbonamide did not exceed the optimal usage (2 wt%) from previous verification [29]. In addition, the *T_dm_*_1_ (294 °C) of the first peak was higher than the *T_dm_* of azodicarbonamide (222 °C) (Figure S2). In this regard, it should not be from the decomposition of the residual blowing agent. Our previous study found that amino (-NH_2_) groups in azodicarbonamide could copolymerize with oxazine rings in PN resin and result in an azo-containing intermediate (Figure 1c) [29]. In addition, it has been reported that azo-containing compounds are thermally active, and the thermal stabilities of azo-containing linear polymers are limited to 200–350 °C [47,48,49], conforming to the *T_dm_*_1_ (294 °C) of this work. Furthermore, as the temperature increased, there was a continuous exothermic phenomenon in the DSC curves of original the PN foam due to the decomposition of the azo-containing section in the PN foam (Figure S3). However, no obvious enthalpy change was observed in the PN foam after thermal resentment at 300 °C (Figure S3). According to these results, the first small peak was primarily from the thermal pyrolysis of the azo-containing copolymerization intermediate. On the other hand, the existence of the closed bubbles in the PN foam should be taken into consideration according to the SEM observations (Figure 5). With increasing temperature, thermal movements of the volatiles resulted from intermediate decomposition, and the gases sealed in the closed cells during the foaming process became violent. When the internal pressure was beyond the critical pressure of the closed bubbles, these gases could break through from the closed cells in the form of weight loss. That is, the first peak is a superposition result of gases from decomposition and foaming. In this regard, it can be concluded that the closed cells have a large effect on the thermal degradation behavior of PN foam. Even for F1000, closed cells could be observed (Figure 5). It can be thus inferred that gas superposition possibly exists in the stages of backbone pyrolysis and further carbonization. In this sense, special attention should be paid to this phenomenon when analyzing the possible volatiles released from the thermal degradation of PN foam.

### 3.3. Monitoring Thermal Degradation of PN Foam by TGA–FTIR

**Figure 7:**
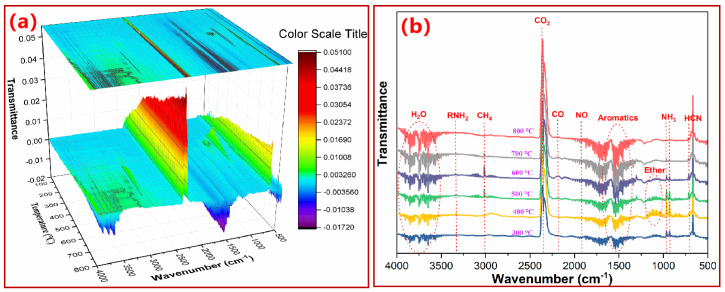
(**a**) 3D TGA–FTIR and (**b**) 2D FTIR spectra of original PN foam (F200).

TGA–FTIR was then utilized to monitor the gases during the thermal degradation of PN foam, as shown in Figure 7. The most remarkable band in Figure 7a is collocated at 2362 cm^−1^, indicating the continuous discharge of CO_2_ (C=O, asymmetric stretching vibration [33]) during PN foam decomposition. From the 2D FTIR spectra, some other volatiles could be detected, as shown in Figure 7b. The peaks from 4000 cm^−1^ to 3400 cm^−1^ correspond to the bending vibration of O-H from hydroxyl compounds, such as H_2_O [35]. The bands between 1500 cm^−1^ and 1200 cm^−1^ are reflections of the stretching vibration of C=C from aromatics [36]. In general, CO_2_, H_2_O, and aromatics could be detected in each thermal degradation stage of the PN foam. However, other volatiles were only recognized at specific temperatures. As per the Lambert–Beer law, the peak intensity is in proportion to the volatile concentration [50]. Herein, the variation in volatile intensity in Figure 7b reflects its evolution process and thus provides bond cleavage information of the PN molecular structure during pyrolysis. 

Near *T_dm_*_1_ (300 °C), apart from CO_2_, H_2_O, and aromatic gases, the peaks at 967 cm^−1^ and 931 cm^−1^ (N-H bending vibration) indicate the release of NH_3_ in the first pyrolysis stage of the PN foam [35]. However, the final curing products of the PN resin were N-containing aromatic heterocycles, which showed high thermal stabilities. In addition, no primary amine (NH_2_) groups were pendant in these heterocycles. NH_3_ should not be generated from the final curing products. On the other hand, it has been reported that NH_3_ is one of the decomposition products of azo-containing compounds [47,48,49]. In addition, some volatile gases, including NH_3_, CO_2_, H_2_O, etc., were produced during the foaming process. Combined with the SEM observations, due to the existence of closed pores, the above gases can be trapped in PN foams. Herein, the production of NH_3_ at 300 °C was not only from the degradation of azo-containing intermediates but also from the release of gases trapped in closed cells in the foaming process, consistent with the TGA/DTG results.

Around *T_dm_*_2_ (400 °C), the broad band at 3012 cm^−1^ of C-H stretching reflects the breakage of methyl (-CH_3_) in the bisphenol A-based molecular structure of PN foam [35,36,37]. The intensive peaks at 1120 cm^−1^ are assigned to the stretching vibration of C-O-C in ethers [33], which resulted from the cleavages of O-containing bonds in the PN foam. The weak absorption at 714 cm^−1^ reveals the escape of HCN by the fragmentation of unreacted Ar-CN bonds [35,36,37]. Notably, the intensity of NH_3_ was the strongest at this time in comparison with other temperatures, as shown in Figure 3b, suggesting the beginning of the decomposition of the tertiary amine (NR_3_) section from the ring-opening polymerization of oxazine. These results indicate that the initial decomposition of the polymer body starts from the dissociation of weak segments in the PN molecular structure at 400 °C.

Close to *T_dm_*_3_ (500 °C), the intensities of CH_4_ (at 3017 cm^−1^ and 1306 cm^−1^) and HCN (at 714 cm^−1^) were enhanced, indicating a continuing breaking of the PN molecular chain. The absorption of NH_3_ was weakened, but meanwhile, a characteristic peak at 3334 cm^−1^ corresponding to amine (RNH_2_) appeared [29,30]. These results indicate the decomposition of N-containing aromatic heterocycles in PN foam is mainly in the form of amine (RNH_2_). In addition, the peak of CO at 2184 cm^−1^ [35,36] indicated an upcoming carbonization stage. At *T_dm_*_3_ (600 °C), the absorption of CO was further enhanced. CH_4_ showed the sharpest peak in comparison with other temperatures, suggesting that CH_4_ was the major volatile during the carbonization stage. With the continuous carbonization to 800 °C, characteristic peaks of RNH_2_, CH_4_, CO, and NH_3_ nearly disappeared, indicating a gradual accomplishment of graphitization. 

In summary, CO_2_, H_2_O, NH_3_, RNH_2_, CH_4_, CO, HCN, and aromatic gases were the main volatiles during the thermal degradation of the PN foam. The volatiles detected at the first thermal degradation stage (i.e., at 300 °C, CO_2_, H_2_O, NH_3_, and aromatic gases) could be also recognized in the second backbone pyrolysis process (i.e., at 400 °C, CO_2_, H_2_O, NH_3_, aromatics, RNH_2_, CH_4_, HCN, and ethers). These results suggest that some volatiles resulting from the first degradation stage were sealed in closed cells and then released until the cell wall was cracked due to the second polymer backbone pyrolysis. In conclusion, there was a gas superposition phenomenon during the pyrolysis of PN foams due to the barrier effect of the closed cells on the prior volatile.

### 3.4. Monitoring Thermal Degradation of PN Foam by TGA–MS 

**Figure 8:**
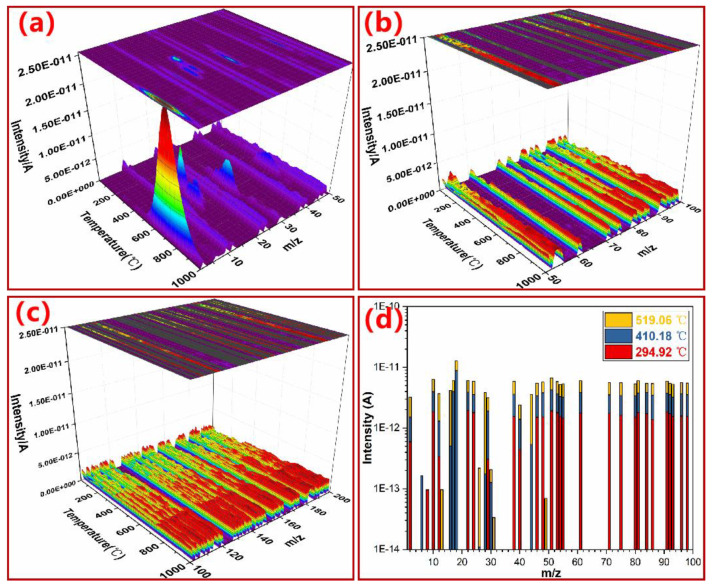
3D ionic current variation for the fragments (**a**) *m*/*z* = 1–50, (**b**) *m*/*z* = 50–100, and (**c**) *m*/*z* = 100–200 and (**d**) ionic intensity at typical temperatures of original PN foam (F200).

Three-dimensional TGA–MS was further employed to confirm the volatiles during the thermal degradation of PN foam, as shown in Figure 8. It can be seen in Figure 8a–c that ionic signals with *m*/*z* < 50 were the most significant, suggesting that the thermal degradation of PN foam mainly gives off small molecule compounds, in line with the major volatiles recognized in the TGA–FTIR results. Under the full scanning mode, the intensity of ionic fragments was strengthened as the temperature increased, indicating that polymer body decomposition and carbonization are the main thermal degradation routes of PN foam.

**Figure 9:**
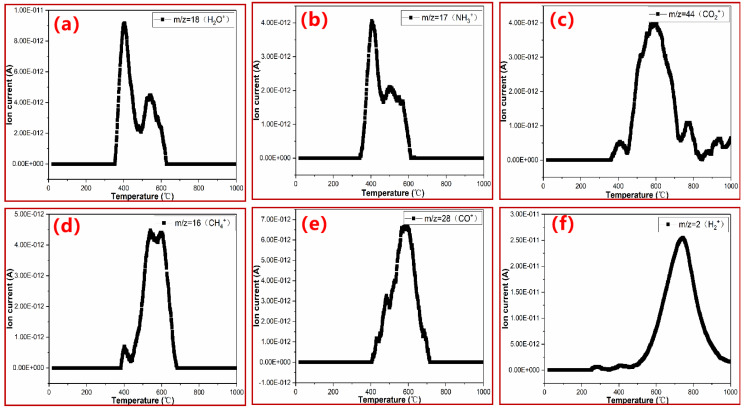
Ionic intensity variation for the fragment of the main gaseous volatile of original PN foam (F200): (**a**) *m*/*z* = 18 (H_2_O^+^), (**b**) *m*/*z* = 17 (NH_3_^+^), (**c**) *m*/*z* = 44 (CO_2_^+^), (**d**) *m*/*z* = 16 (CH_4_^+^), (**e**) *m*/*z* = 28 (CO^+^) and (**f**) *m*/*z* = 2 (H_2_^+^).

Under the selected ion recording mode, the evolution process of major volatile is given in Figure 9a–f. For convenience, Table 1 also summarizes the release temperature range (*T_r_*) and maximum release temperature (*T_rm_*) of each gaseous product.

As can be observed in Figure 9a–c, the initial *T_r_* of NH_3_^+^ (*m*/*z* = 17), H_2_O^+^ (*m*/*z* = 18), and CO_2_^+^ (*m*/*z* = 44) were all below 400 °C, which are relatively low values in comparison with those of other volatiles. These results indicate that H_2_O, CO_2_, and NH_3_ are the main decomposition products at the first degradation stage of PN foam, agreeing well with the TGA–FTIR results at 300 °C (Figure 7b). In addition, the ionic signal of H_2_O^+^ (*m*/*z* = 18, Figure 9a) showed double peaks, with a *T_rm_* of 406 °C and 540 °C, respectively, which were close to the *T_dm_*s (410 °C and 519 °C) of the TGA/DTG results (Figure 6). These results suggest that H_2_O was also generated by the cleavage of O-containing bonds of the PN foam during the pyrolysis of the polymer backbone and further carbonization stage, as also supported by TGA/FTIR scans (Figure 7). However, its first peak was large, while the second one was small (Figure 9a), suggesting that the backbone pyrolysis mainly contributes to the release of H_2_O. A similar case is the ion of NH_3_^+^ (*m*/*z* = 17, Figure 9b), which was generated by the decomposition of tertiary amine (NR_3_) and aromatic heterocycles in PN foam, respectively (Figure 1d). The ionic signal of CO_2_^+^(*m*/*z* = 44, Figure 9b) could be observed in a wide temperature range, matching its trend in TGA–FTIR (Figure 7). 

As shown in Figure 9d,e, the overall evolution processes of CH_4_^+^ (*m*/*z* = 16) and CO^+^ (*m*/*z* = 28) were very similar. They were generated between 400 °C to 700 °C and peaked at nearly 590 °C, in line with the FTIR intensity at 600 °C (Figure 7b). As analyzed previously, CH_4_ was released mainly by the elimination of methyl (-CH_3_) groups in the PN structure, while CO possibly resulted from various processes, i.e., the cleavage of phenolic hydroxyl (Ar-OH) or aryl ether (Ar-O-Ar) bonds in the PN resin and the hypoxic combustion of other organics [35]. 

It is worth noting that the signal of H_2_^+^ (*m*/*z* = 2) was detected with TGA–MS (Figure 9f) but was hard to recolonize with TGA–FTIR. H_2_ is released from 500 °C to 920 °C with a *T_rm_* up to 744 °C. That is, H_2_ was mainly produced in the carbonization stage of the PN foam. Carbonization, in essence, is a dehydrogenation process [51]. Furthermore, it was found that the intensity of H_2_^+^ (*m*/*z* = 2) was the strongest, as shown in Figure 8a, with the highest order of magnitude up to 10^−11^ A, as shown in Figure 9f. Generally, the thermal pyrolysis of a polymer follows a free radical decomposition mechanism, and H_2_ can be generated by the recombination of two hydrogen radicals [50]. Unlike H_2_^+^, no obvious signal of HCN^+^ (*m*/*z* = 27) could be observed with TGA–MS, but the sharp peak at 714 cm^−1^ in TGA–FTIR (Figure 7b) revealed the escape of HCN during the thermal decomposition of the PN foam. Some research has considered that N species (HCN or -CN group) are the precursors of nitrogen oxides (NO) [52,53], and HCN can be released in the form of NO. This is possible, as reflected by the ionic fragment of NO^+^ (*m*/*z* = 30, Figure S4). Alternatively, the -CN group was possibly eliminated with benzene in the form of phthalonitrile (CN-Ar-CN, *m*/*z* = 128, Figure S5). In addition, it was found that ionic signals of aromatics are varied over the whole scope in TGA–MS (Figure S5), although an increasing trend in intensity could be observed with increasing temperature. 

Table 1 also gives a comparison of the major volatiles observed in the thermal degradation of PN foam and other bulk PN resins [35,36,37]. During thermal degradation, PN foam shows more kinds of gaseous volatiles than bulk PN resin [35,36,37]. On the one hand, the polymer host of PN foam is a kind of bi-functional resin that contains both benzoxazine and phthalonitrile groups (Figure 1a). Apart from the thermal degradation of PN, polybenzoxazine could also contribute to the release of volatile. On the other hand, a special form of the foam leads to these differences. Gases sealed in closed cells from the foaming process can be released during the thermal degradation of PN foam. That is, the gases come from two aspects: foaming and decomposition of the polymer. Another difference is that the ionic signal of the volatile shows two or more *T_rm_*s in PN foam rather than a single *T_rm_* in bulk PN [35,36,37]. In addition, the ionic fragments of aromatics in PN foam are varied in TGA–MS. These differences can be attributed to the gas superposition phenomenon during the pyrolysis of PN foams because of the closed cells. Namely, the thermal degradation mechanisms inside and outside the bubble are different. Outside the closed cell, it is easy for volatiles to escape. Inside the closed cell, however, volatiles generated from the foaming and degradation process are sealed until gases break through the bubble layer. Furthermore, the thickness and surrounding of each closed cell is different. As a result, aromatics are varied in TGA–FTIR and TGA–MS, and multiple *T_rm_*s can be observed in each volatile. To conclude, the existence of the closed bubble shows a great effect on the thermal degradation mechanism of PN resin. 

### 3.5. Possible Thermal Degradation Mechanisms of PN Foam

**Figure 10:**
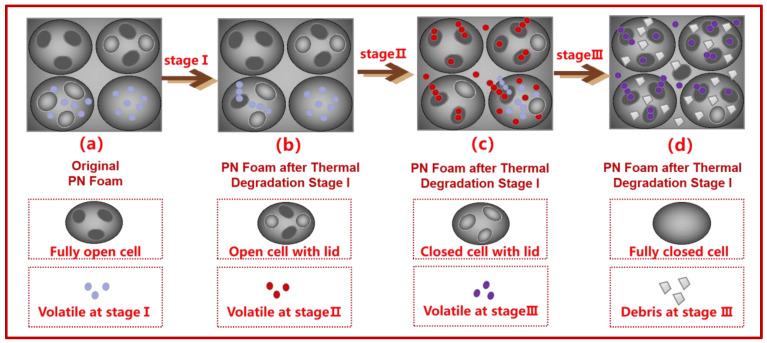
Thermal degradation mechanism of PN foam at the macroscopic level.

According to TGA/DTG results, the thermal degradation of PN foam can be divided into three stages: intermediate transition, backbone decomposition, and carbonization. According to the TGA/FTIR and TGA/MS results, the main volatiles were clarified as CO_2_, H_2_O, NH_3_, RNH_2_, CH_4_, CO, HCN, and aromatic gases. Compared with bulk PN resin, the existence of the closed bubble in the PN foam resulted in more complex thermal degradation behavior. By abstracting the SEM images of the original foam (as illustrated in Figure S1), the structure of the PN foam could be simplified into Figure 10a. Bubbles in PN foam can be classified into four types: fully open cell, open cell with lid, closed cell with lid, and fully closed cell. The existence of bubbles had a large effect on its thermal degradation process. Combined with these findings, the possible thermal degradation mechanism of PN foam at each stage can be proposed from the aspects of the macroscopic and macromolecular levels, as follows. 

**Figure 11:**
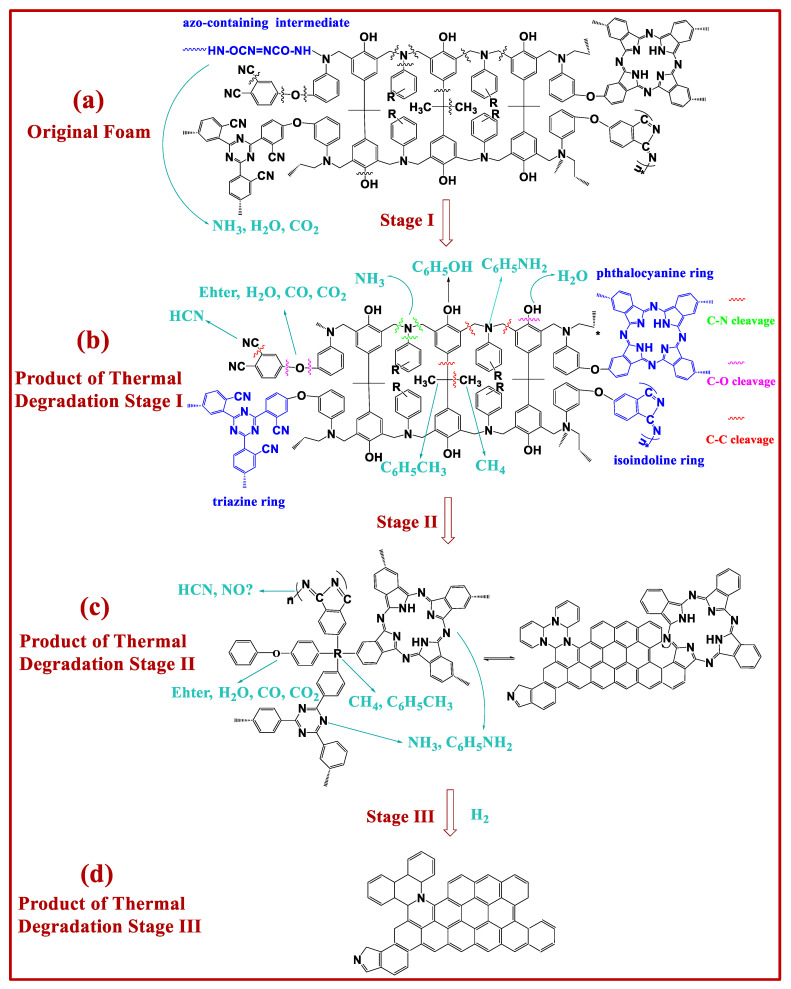
Thermal degradation mechanism of PN foam at the molecular level.

*Stage I*. As the temperature increased to around 294 °C, the thermal movement of the volatiles sealed in the closed cells became more and more violent until they broke through from the weak region of the closed cells (Figure 10b). On the one hand, some of the volatiles originated from the foaming process. On the other hand, the azo-containing copolymerization intermediate could decompose at this time by releasing NH_3_, H_2_O, and CO_2_ (Figure 11a). 

*Stage II*. The pyrolysis of the polymer backbone started when the temperature continuously rose to 410 °C. That is, the polymer on the inner and outer walls of the bubbles decomposed. In fully closed cells, apart from the volatiles trapped during the foaming process, the decomposition of the resin from the inner surface of the bubble produced new species of volatiles. A superposition of old and new volatiles may break through the weak region of the fully closed cell surface (Figure 10c). Therefore, the volatiles in stage II are much more complicated. The order of covalent bond energy in PN foam is C-N ≈ C-O ≈ C-C < N-H < C-H < O-H < C=C < C=N < C=O < C≡N [54] (Table S3). Herein, the prior cleavage started from weak bonds of C-N, C-O, and C-C in the aliphatic chain of the PN molecular structure (Figure 11b,c), resulting in highly aromatic products with the release of H_2_O, CO_2_, NH_3_, CO, CH_4_, RNH_2_, HCN, and aromatic gases.

*Stage III*. The decomposition of highly aromatic products happened in ether, triazine, and isoindole with less thermal stability, finally resulting in the hybrid graphite by releasing a series of volatiles [37] (Figure 11d). A gas superposition and diffusion made the closed cells open, and the debris carbonization products were deposited in the PN foam (Figure 10d). 

## 4. Conclusions

In this study, thermal degradation behaviors and the corresponding mechanisms of high-temperature-resistant PN foam were systematically investigated at the macroscopic and molecular levels. It was found that the thermal degradation behavior of PN foam is different from that of bulky PN resin. During the foam pyrolysis process, there was a gas superposition phenomenon as the release of the internal volatile was retarded by the closed cells in the PN foam. PN foam mainly involves a three-stage thermal degradation mechanism. The first degradation mainly released gases such as H_2_O, CO_2_, and NH_3_, which were generated from azo-containing intermediate decomposition and the trapped gases in the closed cells during the foaming process. The second was the major decomposition of the polymer body that started from the cleavage of C-N, C-C, and C-O in the aliphatic chain, resulting in highly aromatic products by releasing H_2_O, CO_2_, NH_3_, CO, CH_4_, RNH_2_, HCN, and aromatic gases. The third carbonization process led to a N hybrid graphite, causing flour-like carbonation debris to be deposited inside the foam. It should be addressed from the current findings that the sample form of a polymer can have a large effect on its thermal degradation behavior. In view of the excellent thermal resistance of PN foam, future research can focus on the further functionalization and applications of these foams in extreme environments.

## Figures and Tables

**Table 1:** A comparison of the major ionic fragments of PN foam and bulk PN resin.

Gaseous Volatile	*m*/*z*	*T_r_* of Gaseous Product (°C)	*T_rm_* of Benzoxazine-Containing PN Foam (°C)	*T_rm_* of Resorcinol-Based PN Resin (°C) [35]	*T_rm_* of l-Tyrosine-Based PN Resin (°C) [36]	*T_rm_* of Adenine-Containing PN Resin (°C) [37]
H_2_O	18	340–620	406, 540	560	550	Not given
NH_3_	17	350–810	403, 501	580	566	varied
CO_2_	44	360–840	407, 595, 776	550	437	Not given
CH_4_	16	400–670	403, 539, 601	590	444	590, 890
CO	28	400–720	483, 590	590, 720	439	Not given
H_2_	2	500–920	744	Not given	766	Not given
HCN/NO?	27/30	380–1000	403, 561, 766	HCN, 600	HCN, 576	HCN, 600
Ar-R	>78	varied	varied	C_6_H_6_, 590	Not given	500

## Data Availability

Data are contained within the article.

## Supplementary Materials

The following supporting information can be downloaded at: https://www.mdpi.com/article/10.3390/polym15193947/s1, Table S1: Main information of PN foam in Raman spectra; Table S2: FWHM values of PN foam on d_002_; Table S3: bond energy of covalent bond in PN foam; Figure S1: Bubble types in PN foams; Figure S2: TGA/DTG curves of azodicarbonamide; Figure S3: DSC curve of (a) original PN foam and (b) PN foam after after thermal treatment at 300 °C/1h; Figure S4: The ionic signal of NO; Figure S5: The ionic signals of aromatics.

## References

[1] Derradji M., Wang J., Liu W. (2018). Phthalonitrile Resins and Composites.

[2] Ye J., Zhang S., Wu M., Liu X., Liu X. (2023). Thermal, mechanical and dielectric property enhancement of benzoxazine-containing phthalonitrile resin: The effect of functional oligomeric polyphenyl ether. Polymer.

[3] Chen Z., Wang L., Lin J., Du L. (2021). A theoretical insight into the curing mechanism of phthalonitrile resins promoted by aromatic amines. Phys. Chem. Chem. Phys..

[4] Yang X., Lei W., Liu Q., Li Y., Li K., Wang P., Feng W. (2021). A tailor-made method to recycle slow-curing thermoset of phthalonitrile by constructing self-composite with the improved properties. Compos. Commun..

[5] Xu M., Ren D., Chen L., Li K., Liu X. (2018). Understanding of the polymerization mechanism of the phthalonitrile-based resins containing benzoxazine and their thermal stability. Polymer.

[6] Wang Z., Yang X., Wei J., Xu M., Tong L., Zhao R., Liu X. (2012). Morphological, electrical, thermal and mechanical properties of phthalocyanine/multi-wall carbon nanotubes nanocomposites prepared by masterbatch dilution. J. Polym. Res..

[7] Fu T., Zhao X., Chen L., Fu T., Zhao X., Chen L., Wu W., Zhao Q., Wang X., Guo D. (2019). Bioinspired color changing molecular sensor toward early fire detection based on transformation of phthalonitrile to phthalocyanine. Adv. Funct. Mater..

[8] Xu X., Xu M., Liu T., Ren D., Liu X. (2022). Understanding the curing behaviors and properties of phthalonitrile containing benzoxazine with a new type of aniline curing agent. Polym. Test..

[9] Bai S., Sun X., Wu M., Shi X., Chen X., Yu X., Zhang Q. (2020). Effects of pure and intercalated halloysites on thermal properties of phthalonitrile resin nanocomposites. Polym. Degrad. Stab..

[10] Chen X., Cai Y., Qu X., Chen J., Zheng D. (2022). Preparation of a self-catalyzed amino-epoxy phthalonitrile resin with a large processing window. J. Mater. Sci..

[11] Kolesnikov T.I., Orlova A.M., Tsegelskaya A.Y., Cherkaev G.V., Kechekyan A.S., Buzin A.I., Dmitryakov P.V., Belousov S.I., Abramov I.G., Serushkina O.V. (2021). Dual-curing propargyl-phthalonitrile imide-based thermoset: Synthesis, characterization and curing behavior. Eur. Polym. J..

[12] Yakovlev M.V., Morozov O.S., Afanaseva E.S., Babkin A.V., Kepman A.V. (2020). Tri-functional phthalonitrile monomer as stiffness increasing additive for easy processable high performance resins. React. Funct. Polym..

[13] Chaussoy N., Brandt D., Gérard J.F. (2023). Phthalonitrile functionalized resoles—Use of 2,3-dicyanohydroquinone as a versatile monomer for resins with very high thermal stability. Polym. Degrad. Stab..

[14] Nechausov S., Aleksanova A., Morozov O., Babkin A., Kepman A., Avdeev V., Bulgakov B. (2022). Heat-Resistant Phthalonitrile-Based Resins for 3D Printing via Vat Photopolymerization. ACS Appl. Polym. Mater..

[15] Bulgakov B.A., Sulimov A.V., Babkin A.V., Timoshkin I.A., Solopchenko A.V., Kepman A.V., Avdeev V.V. (2017). Phthalonitrile-carbon fiber composites produced by vacuum infusion process. J. Compos. Mater..

[16] Yang X., Li K., Xu M., Jia K., Liu X. (2017). Designing a low-temperature curable phenolic/benzoxazine-functionalized phthalonitrile copolymers for high performance composite laminates. J. Polym. Res..

[17] Bulgakov B.A., Morozov O.S., Timoshkin I.A., Babkin A.V., Kepman A.V. (2021). Bisphthalonitrile-based thermosets as heat-resistant matrices for fiber reinforced plastics. Polym. Sci. Ser. C.

[18] Derradji M., Wang J., Liu W. (2016). High performance ceramic-based phthalonitrile micro and nanocomposites. Mater. Lett..

[19] Liu C., Qiao Y., Jia H., Li N., Chen Y., Jian X. (2021). Improved mechanical properties of basalt fiber/phthalonitrile composites modified by thermoplastic poly (phthalazinone ether nitrile)s. Polymer.

[20] Yang X., Li K., Xu M., Liu X. (2018). Significant improvement of thermal oxidative mechanical properties in phthalonitrile GFRP composites by introducing microsilica as complementary reinforcement. Compos. Part B Eng..

[21] Sun B.G., Shi H.Q., Yang K.X., Lei Q., Li Y.Q., Fu Y.Q., Hu N., Guo Y., Zhou H., Fu S.Y. (2020). Effects of 3-aminophenylacetylene on mechanical properties at elevated temperatures of carbon fiber/phthalonitrile composites. Compos. Commun..

[22] Sun B.G., Lei Q., Guo Y., Shi H.Q., Sun J.B., Yang K.X., Zhou H., Li Y.Q., Hu N., Wang H. (2019). Enhanced mechanical properties at 400 °C of carbon fabric reinforced phthalonitrile composites by high temperature postcure. Compos. Part B Eng..

[23] Derradji M., Ramdani N., Zhang T., Wang J., Gong L., Xu X., Lin Z., Henniche A., Rahoma H., Liu W. (2016). Effect of silane surface modified titania nanoparticles on the thermal, mechanical, and corrosion protective properties of a bisphenol-A based phthalonitrile resin. Prog. Org. Coat..

[24] Laskoski M., Keller T.M., Qadri S.B. (2007). Direct conversion of highly aromatic phthalonitrile thermosetting resins into carbon nanotube containing solids. Polymer.

[25] Lei Y., Zhao R., Xu M., Zhao X., Yang X., Guo H., Zhong J., Liu X. (2012). Production of empty and iron-filled multiwalled carbon nanotubes from iron–phthalocyanine polymer and their electromagnetic properties. J. Mater. Sci. Mater. Electron..

[26] Weng Z., Zhang K., Qi Y., Zhang T., Xia M., Hu F., Zhang S., Liu C., Wang J., Jian X. (2020). Scalable fabrication of heteroatom-doped versatile hierarchical porous carbons with an all-in-one phthalonitrile precursor and their applications. Carbon.

[27] Zeng J., Xie W., Zhou H., Zhao T., Xu B., Jiang Q., Algadi H., Zhou Z., Gu H. (2023). Nitrogen-doped graphite-like carbon derived from phthalonitrile resin with controllable negative magnetoresistance and negative permittivity. Adv. Compos. Hybrid Mater..

[28] Zhang L., Liu M., Roy S., Chu E., See K., Hu X. (2016). Phthalonitrile-based carbon foam with high specific mechanical strength and superior electromagnetic interference shielding performance. ACS Appl. Mater. Interfaces.

[29] Lei W., Wang D., Li Y., Li K., Liu Q., Wang P., Feng W., Liu Q., Yang X. (2022). High temperature resistant polymer foam based on bi-functional benzoxazine-phthalonitrile resin. Polym. Degrad. Stab..

[30] Lei W., Wang D., Liu Q., Li K., Li Y., Zhong F., Liu Q., Wang P., Feng W., Yang X. (2022). Large-scale preparation of uniform millet bread-like durable benzoxazine-phthalonitrile foam with outstanding mechanical and thermal properties. Polymers.

[31] Tay Y.S., Liu M., Lim J.S.K., Chen H., Hu X. (2020). Phthalonitrile prepolymer and PAN blends: New strategy for precursor stabilization and pyrolytic char yield enhancement. Polym. Degrad. Stab..

[32] Mortezaeikia V., Tavakoli O., Khodaparasti M.S. (2021). A review on kinetic study approach for pyrolysis of plastic wastes using thermogravimetric analysis. J. Anal. Appl. Pyrolysis.

[33] Yang X., Li Y., Lei W., Liu X., Zeng Q., Liu Q., Feng W., Li K., Wang P. (2021). Thermal degradation behaviors of poly (arylene ether nitrile) bearing pendant carboxyl groups. Polym. Degrad. Stab..

[34] Liang B., Hu J., Yuan P., Li C., Li R., Liu Y., Zeng K., Yang G. (2019). Kinetics of the pyrolysis process of phthalonitrile resin. Thermochim. Acta.

[35] Liang B., Wang J., Hu J., Li C., Li R., Liu Y., Zeng K., Yang G. (2019). TG-MS-FTIR study on pyrolysis behavior of phthalonitrile resin. Polym. Degrad. Stab..

[36] Zhou T., Xiao H., Peng W., Liang B., Liu Y., Lv J., Hu J., Zeng K., Yang G. (2020). Study on pyrolysis behaviors of L-tyrosine-based phthalonitrile resin. Polym. Test..

[37] Guo X., Liang B., Chen M., He X., Xiao H., Zeng K., Zhou T., Hu J., Yang G. (2021). Study on pyrolysis behavior of bio-based adenine containing phthalonitrile resin obtained by powder metallurgy-like process. Polym. Degrad. Stab..

[38] Lobanova M.S., Aleshkevich V.V., Yablokova M.Y., Morozov O.S., Babkin A.V., Kepman A.V., Avdeev V.V., Bulgakov B.A. (2023). Kinetics of the oxidative aging of phthalonitrile resins and their effects on the mechanical properties of thermosets. Thermochim. Acta.

[39] Jin F.L., Zhao M., Park M., Park S. (2019). Recent trends of foaming in polymer processing: A review. Polymers.

[40] Fraleoni-Morgera A., Chhikara M. (2019). Polymer-based nano-composites for thermal insulation. Adv. Eng. Mater..

[41] Członka S., Strąkowska A., Kairytė A. (2020). Effect of walnut shells and silanized walnut shells on the mechanical and thermal properties of rigid polyurethane foams. Polym. Test..

[42] Jiao L., Xiao H., Wang Q., Sun J. (2013). Thermal degradation characteristics of rigid polyurethane foam and the volatile products analysis with TG-FTIR-MS. Polym. Degrad. Stab..

[43] (2006). Cellular Plastics and Rubbers—Determination of Apparent Density.

[44] Han M., Yin X., Ren S., Duan W., Zhang L., Cheng L. (2016). Core/shell structured C/ZnO nanoparticles composites for effective electromagnetic wave absorption. RSC Adv..

[45] Naskar A., Paul S. (2022). Non-destructive measurement of grinding-induced deformation-depth using grazing incidence X-ray diffraction technique. NDT E Int..

[46] Ferrari A.C., Kleinsorge B., Morrison N.A., Hart A., Stolojan V., Robertson J. (1999). Stress reduction and bond stability during thermal annealing of tetrahedral amorphous carbon. J. Appl. Phys..

[47] Zhou D.W., Liang L.Y., Lu M.G. (2011). Dimeric liquid crystalline thermosets from azo-containing diglycidyl ether cured by anhydride. Polym. Bull..

[48] Ayer M.A., Simon Y.C., Weder C. (2016). Azo-containing polymers with degradation on-demand feature. Macromolecules.

[49] Lisa G., Păiuş C., Raicu-Luca A., Hurduc N. (2012). Azo-polysiloxanes thermal stability study: Thermal stability of azo-polysiloxanes with biological applications. High Perform. Polym..

[50] Kumazawa H., Inoue M., Kasuya T. (2003). Photocatalytic degradation of volatile and nonvolatile organic compounds on titanium dioxide particles using fluidized beds. Ind. Eng. Chem. Res..

[51] Benhammada A., Trache D. (2020). Thermal decomposition of energetic materials using TG-FTIR and TG-MS: A state-of-the-art review. Appl. Spectrosc. Rev..

[52] Bai J., Yu C., Li L., Wu P., Luo Z., Ni M. (2013). Experimental study on the NO and N_2_O formation characteristics during biomass combustion. Energy Fuels.

[53] Yang S., Zhu X., Wang J., Jin X., Liu Y., Qian F., Zhang S., Chen J. (2015). Combustion of hazardous biological waste derived from the fermentation of antibiotics using TG–FTIR and Py–GC/MS techniques. Bioresour. Technol..

[54] Sanderson R. (2012). Chemical Bonds and Bonds Energy.

