# Supplementary material for: Understanding the Thermal Degradation Mechanism of High-Temperature-Resistant Phthalonitrile Foam at Macroscopic and Molecular Levels
Source: Polymers (Basel). 2023 Sep 29;15(19):3947. doi: 10.3390/polym15193947 (PMC10575044; doi:10.3390/polym15193947)
Supplement: Supplementary file 1 [file polymers-15-03947-s001.zip › polymers-2602860-supplementary.pdf]

*Supporting information for*

# Understanding the thermal degradation mechanism of high-temperature-resistant phthalonitrile foam at macroscopic and molecular levels

Xulin Yang[1,2,3*], Yi Li[1], Wenwu Lei[1,4], Zhongxiang Bai[3], Yingqing Zhan[5], Ying Li[1], Kui Li[1], Pan Wang[1], Wei Feng[1], Qi Liu[2*]

*1 School of Mechanical Engineering, Chengdu University, Chengdu, 610106, P. R. China*

*2 Chemical Synthesis and Pollution Control Key Laboratory of Sichuan Province, China West Normal University, Nanchong, 637009, P. R. China*

*3 Institute for Advanced Study, Chengdu University, Chengdu 610106, China*

*4 Chengdu Hongbo Industrial Co.,Ltd., Chengdu,610199,China*

*5 College of Chemistry and Chemical Engineering, Southwest Petroleum University, Chengdu,610500, P. R. China*

Email address:
*yangxulin@cdu.edu.cn*
*liuqi@cwnu.edu.cn*

**Content:**

**Table. S1 Main information of PN foam in Raman spectra**
**Table. S2 FWHM values of PN foam on $d_{002}$**
**Table S3 bond energy of covalent bond in PN foam**

**Figure. S1. Bubble types in PN foams**
**Figure. S2 TGA/DTG curves of azodicarbonamide**
**Figure. S3. DSC curve of (a) original PN foam and (b) PN foam after after thermal treatment at 300 ℃/1h**
**Figure. S4 The ionic signal of NO**
**Figure. S5 The ionic signals of aromatics**

* Corresponding author. E-mail address: yangxulin@cdu.edu.cn
* Corresponding author. E-mail address: liuqi@cwnu.edu.cn

**Table. S1 Main information of PN foam in Raman spectra**

| Sample | F200 | F400 | F600 | F800 | F1000 |
|---|---|---|---|---|---|
| $I_D/I_G$ | 0.8441 | 0.8439 | 0.8061 | 0.9669 | 0.9996 |
| D band ($cm^{-1}$) | 1352.4 | 1352.4 | 1348.5 | 1333.1 | 1336.9 |
| G band ($cm^{-1}$) | 1564.5 | 1572.3 | 1583.8 | 1585.7 | 1591.5 |
| 2D band ($cm^{-1}$) | 2843.1 | 2779.4 | 2825.7 | 2862.3 | 2852.7 |

**Table. S2 FWHM values of PN foam on $d_{002}$**

| Sample | F200 | F400 | F600 | F800 | F1000 |
| --- | --- | --- | --- | --- | --- |
| FWHM | 6.77 | 11.38 | 14.35 | 16.07 | 17.26 |

**Table S3 bond energy of covalent bond in PN foam [1]**

| Bond type | C-N | C-O | C-C | N-H | C-H | O-H | C=C | C=N | C=O | C≡N |
|---|---|---|---|---|---|---|---|---|---|---|
| **Bond energy (kJ/mol)** | 305 | 326 | 332 | 391 | 414 | 464 | 611 | 615 | 728 | 891 |

## Reference

[1] Sanderson R. Chemical bonds and bonds energy. Elsevier, 2012.

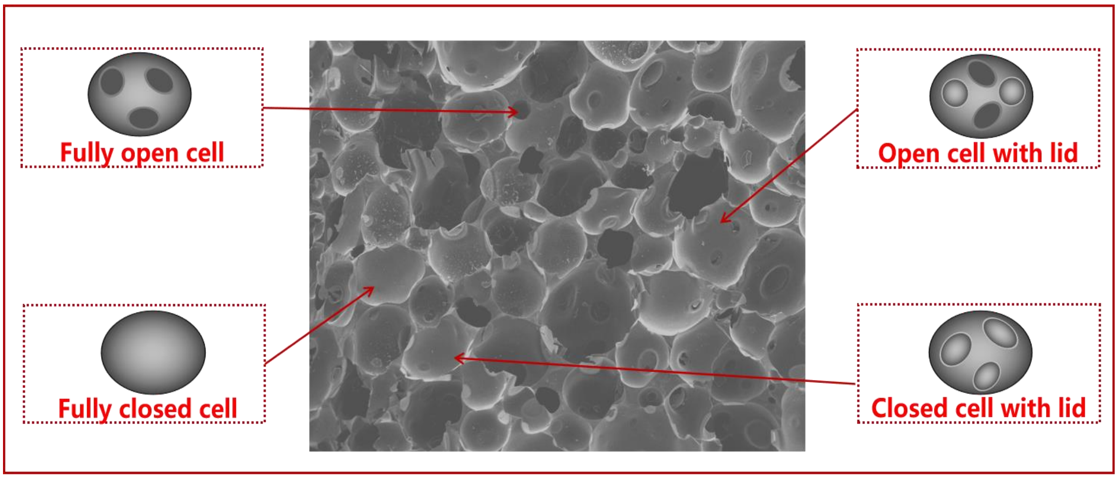


**Figure. S1. Bubble types in PN foams**

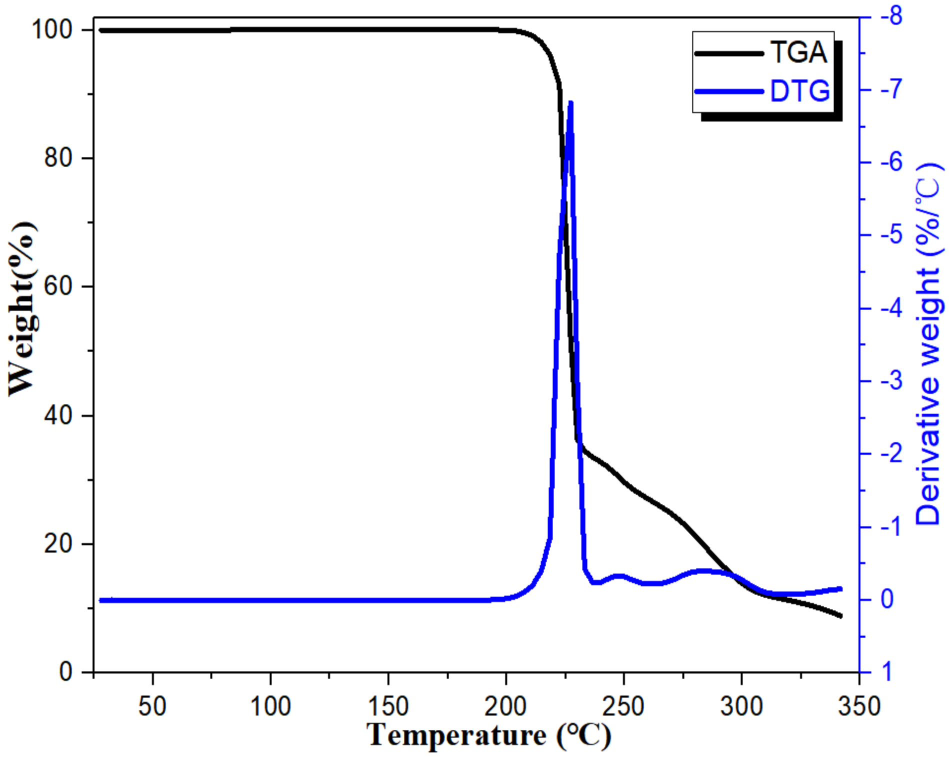


**Figure. S2 TGA/DTG curves of azodicarbonamide**

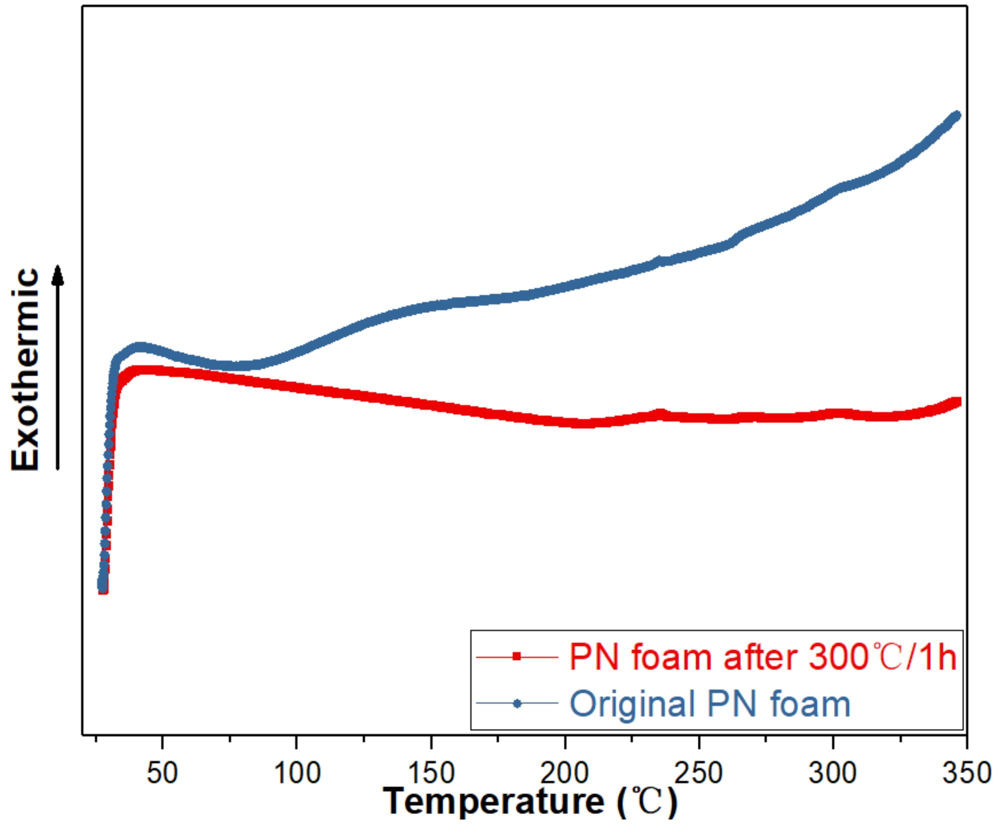


**Figure. S3. DSC curve of (a) original PN foam and (b) PN foam after after thermal treatment at 300 °C/1h**

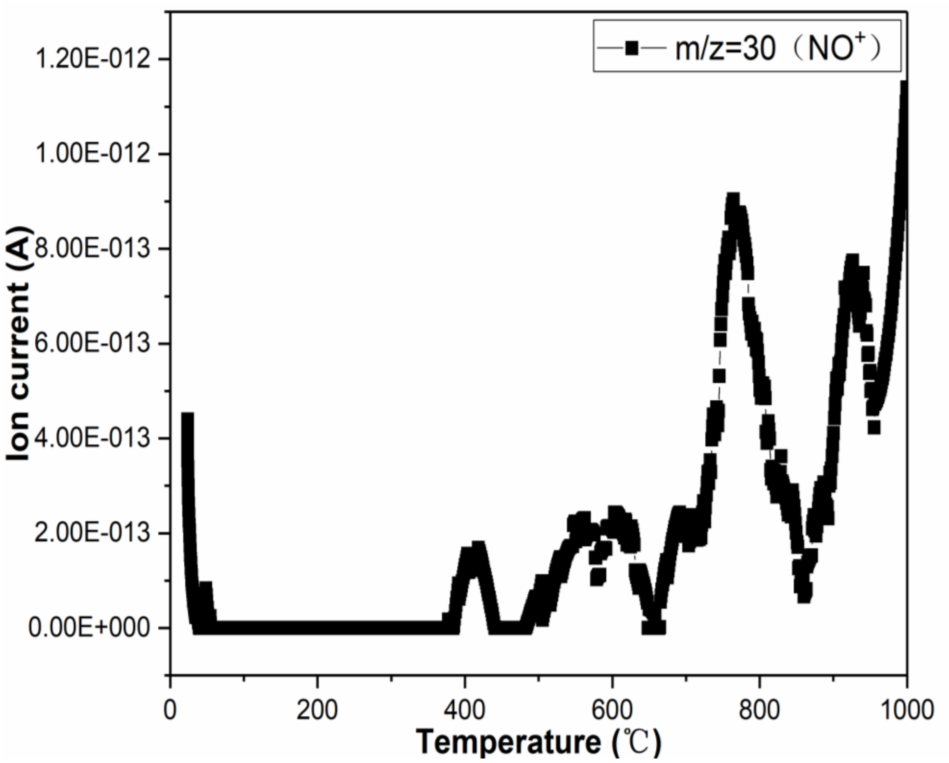


**Figure. S4 The ionic signal of NO**

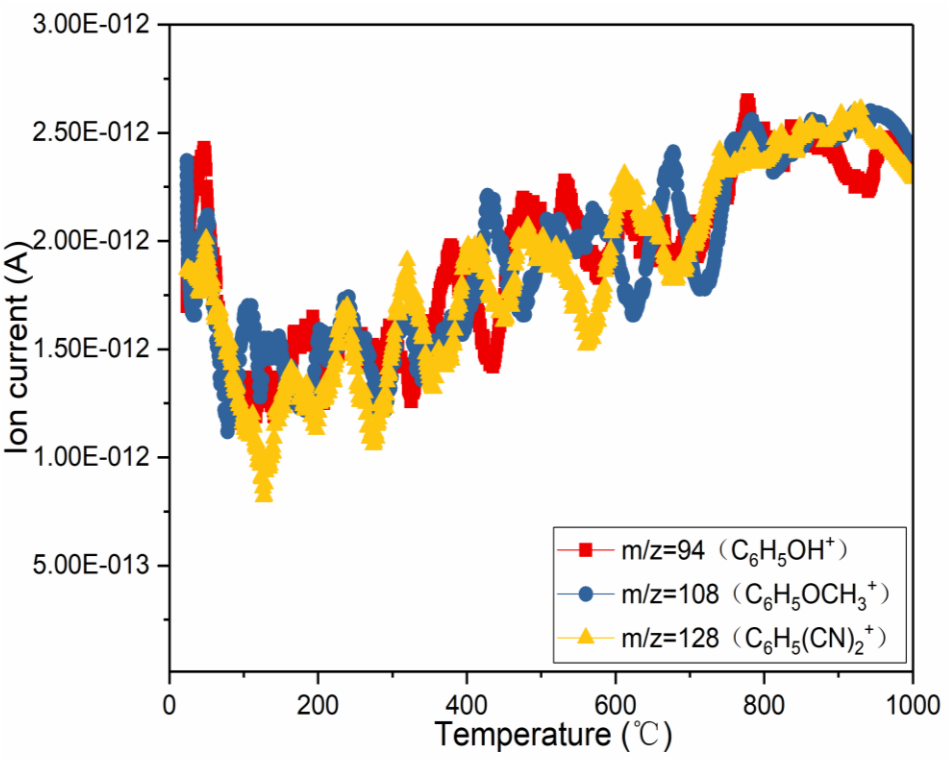


**Figure. S5 The ionic signals of aromatics**
